# A thematic analysis of the introduction of smart-hub technology to a rural Psychiatry of Old Age Service during Covid-19 lockdowns

**DOI:** 10.1192/j.eurpsy.2024.188

**Published:** 2024-08-27

**Authors:** S. Patel, A. Gannon, M. Cryan, C. Dolan, C. McDonald, C. Houghton, G. McCarthy

**Affiliations:** ^1^Liaison Psychiatry, University Hospital Galway; ^2^School Of Medicine, University Of Galway, Galway; ^3^Sligo Leitrim Mental Health Services, Sligo, Ireland; ^4^Psychiatry of Old Age, Sligo Leitrim Mental Health Services, Sligo; ^5^Psychiatry, University Hospital Galway; ^6^School of Nursing and Midwifery, University Of Galway, Galway; ^7^School Of Medicine, University of Galway, Sligo, Ireland

## Abstract

**Introduction:**

The use of smart technology in supporting older adults is a growing field of research. However, there is little qualitative research on the experiences of patients utilizing this technology, particularly those attending psychiatry services.

**Objectives:**

To explore the experiences of staff and patients utilizing smart-hubs implemented during the Covid-19 pandemic to provide remote audio/visual communication and smart AI personal assistant technology for the management of patients in a rural Psychiatry of Old Age service.

**Methods:**

Smart hubs were installed in patient homes and in the Psychiatry of Old Age base during the Covid-19 pandemic when lockdown restrictions limited in-person service provision. Patients and staff utilized the smart hubs for its assistive technology and to engage with each other. Semi-structured qualitative interviews were conducted of 10 staff and 15 patients at 6-12 months following the introduction of the smart hubs and thematic analysis was conducted to generate themes.

**Results:**

Three themes were generated from the thematic analysis: 1) Openness to SMART hub technology, 2) Getting set-up and 3) Keeping SMART. The majority of participants did not have much experience using smart technology prior to the intervention. However, many participants reported that they would be comfortable using technology. The Covid-19 pandemic contributed to the rapid adoption of this intervention within the service with mixed views regarding the smart hub prior to implementation. The role of key individuals such as staff and family was highlighted in supporting older persons with setting-up the smart hub. Technical needs included the need for a strong internet connection and technical limitations were driven by privacy, cost and regional considerations. Many patients were able to utilize the smart hub independently to access interests, therapeutic activities and as a memory aid. The smart hub offered a novel way to connect to services and families and was also seen as a companion by some patients and staff to help address loneliness and isolation. The majority of participants found the use of smart hubs acceptable and were willing to utilize the smart hub in the future as an adjunct to face to face psychiatric interventions. However, suggestions for future use included the need for additional training as users felt that there was more they could do with the smart hub, continued support to manage any challenges and improved information leaflets to better engage users.

**Conclusions:**

Smart hub technology offers an alternate means of providing remote and inclusive psychiatric care to older patients unable to access services in person and at risk of deterioration without intervention in the community.

**Disclosure of Interest:**

None Declared

